# Implementation of a multidisciplinary psychoeducational intervention for Parkinson’s disease patients and carers in the community: study protocol

**DOI:** 10.1186/s12875-018-0730-9

**Published:** 2018-04-05

**Authors:** M. V. Navarta-Sánchez, M. E. Ursua, M. Riverol Fernández, L. Ambrosio, M. Medina, S. Díaz de Cerio, M. J. Álvarez, J. M. Senosiain, A. Gorraiz, N. Caparrós, S. Anaut, R. Martín-Lanas, M. Recio, M. C. Portillo

**Affiliations:** 1 Faculty of Nursing, University of Navarre, C/ Irunlarrea, s/n, Edif. De los Castaños, 31008 Pamplona, Navarre Spain; 2 Primary Health Care Center of San Juan, Navarre Service of Health-Osasunbidea, Plaza Obispo Irurita s/n Planta Baja, 31011 Pamplona, Navarre Spain; 3 Department of Neurology, Clínica Universidad de Navarra, Avenida Pio XII, 36, 31008 Pamplona, Navarre Spain; 4 Navarre Association of Parkinson’s patients, C/ Aralar 17, 31004 Pamplona, Navarre Spain; 5 Public University of Navarre, Campus de Arrosadia, s/n, 31006 Pamplona, Navarre Spain; 6 Faculty of Law and Social Sciences, University of La Rioja, C/ Cigüeña 60, 26004 Logroño, La Rioja Spain; 7 Department of Social Work, Public University of Navarre, Campus de Arrosadia, s/n, 31006 Pamplona, Navarre Spain; 8 Department of Psychiatry and Medical Psychology, Clínica Universidad de Navarra, Avenida Pio XII, 36, 31008 Pamplona, Navarre Spain; 9 NIHR CLAHRC Wessex, Faculty of Health Sciences, University of Southampton Highfield Campus, University Road, Southampton, SO17 1BJ UK

**Keywords:** Coping, Intervention, Long-term conditions, Primary health care, Psychosocial adjustment, Quality of life, Quasi-experimental design

## Abstract

**Background:**

Parkinson’s disease progressively limits patients at different levels and as a result family members play a key role in their care. However, studies show lack of an integrative approach in Primary Care to respond to the difficulties and psychosocial changes experienced by them. The aim of this study is to evaluate the effects of a multidisciplinary psychoeducational intervention focusing on improving coping skills, the psychosocial adjustment to Parkinson’s disease and the quality of life in patients and family carers in a Primary Care setting.

**Methods:**

This quasi-experimental study with control group and mixed methods was designed to evaluate a multidisciplinary psychoeducational intervention. Based on the study power calculations, 100 people with Parkinson’s disease and 100 family carers will be recruited and assigned to two groups. The intervention group will receive the ReNACE psychoeducational intervention. The control group will be given a general educational programme. The study will be carried out in six community-based health centres. The results obtained from the two groups will be collected for evaluation at three time points: at baseline, immediately after the intervention and at 6 months post-intervention. The results will be measured with these instruments: the Quality of Life Scale PDQ-39 for patients and the Scale of Quality of Life of Care-givers SQLC for family carers, and for all participants the Psychosocial Adjustment to Illness scale and the Brief COPE Inventory. Focus groups will be organised with some patients and family carers who will have received the ReNACE psychoeducational intervention and also with the healthcare professionals involved in its development.

**Discussion:**

An important gap exists in the knowledge and application of interventions with a psychosocial approach for people with PD and family carers as a whole. This study will promote this comprehensive approach in Primary Care, which will clearly contribute in the existing knowledge and could reduce the burden of PD for patients and family carers, and also in other long-term conditions.

**Trial registration:**

NCT03129425 (ClinicalTrials.gov). Retrospectively registered on April 26, 2017.

## Background

Parkinson’s disease (PD) is a neurodegenerative disease which affects about 10 million people worldwide [1–3], and which currently has no cure. Numerous studies have been published which show the difficulties and psychosocial changes experienced by people with PD in their lives [4–8]. Similarly, recent research has highlighted that family members caring for people with PD also feel an important impact on their well-being and quality of life [9–11], as they are the patients’ main allies in order for them to carry on the activities of daily life. However, the limited and non-comprehensive approach that is offered to patients with PD and their family carers from Primary Care appears to be insufficient to encourage them to adapt to the changes that they experience as a consequence of this long-term condition (LTC) [12–15]. According to evidence, gender, education, coping, social networks or culture, are social and personal factors that can facilitate or hinder the psychosocial adjustment [16, 17]. In particular, coping has been identified as an essential factor in the improvement of the psychosocial adjustment to different LTCs [16, 17] and also to PD [11, 18]. This is because coping could help the patient with PD and the family carer in their search for balance in their lives [4, 14]. And also due to the psychosocial adaptation to PD is in turn a key mechanism for achieving better outcomes in terms of quality of life of patients with PD and their family carers [11, 18]. At present, few interventions [19, 20] have been found in the literature that aimed to improve coping skills and the psychosocial adjustment to PD. However, surprisingly changes in the development of coping skills and the psychosocial adjustment to PD were not directly measured in those studies [19, 20]. In addition, there is scarce number of psychoeducational interventions [20] that have taken into account needs from dyads, patients with PD and their family carers. Nevertheless, the influence that PD may have on the family carer’s wellbeing [9–11] cannot be neglected by healthcare professionals. To deal with this challenge in clinical practice, it is essential to develop and evaluate psychoeducational interventions which would help to counsel and motivate people with PD and their family carers in the adoption of coping skills which would lead to a better psychosocial adjustment to PD.

## Methods

### Aim

The aim of this present study is to improve the quality of life of people with PD and their family carers by means of a multidisciplinary psychoeducational intervention focusing on fostering coping strategies and their psychosocial adjustment to PD. The study also seeks to compare the effectiveness of the psychoeducational intervention focusing on the acquisition of coping skills with a control group receiving a general educational programme. The study will evaluate the perceptions, opinions and satisfaction of the patients and family carers who receive the psychoeducational intervention and will explore the reflections of the social and healthcare providers involved in this intervention.

On the basis of previous research [9–14, 18], the hypothesis we propose is that through the development of coping skills which will contribute to promoting a positive psychosocial adjustment to PD, the quality of life of the people with PD and their family carers will be improved. And the second hypothesis we suggest is that this psychoeducational intervention will enhance the quality of life of the patients and family carers more than a general education programme.

If we find significant results in this psychoeducational approach, we consider that at the long-term, this could develop larger scale interventions and have positive effects on the overall health of patients and their family carers. In particular, we expect that, by integrating psychoeducational interventions into care pathways, the patients and family carers will perceive a greater sense of normality in their lives [4, 10, 12, 21], which will also promote compliance with drug treatments and healthy lifestyles and professionals would be able to identify and support cases with poor illness management and coordinate levels of care more efficiently.

### Design

**Fig. 1:**
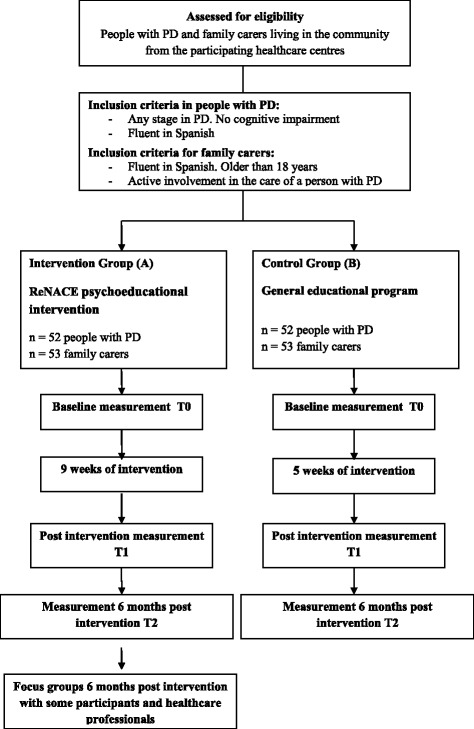
Flow diagram with the design of this study. PD: Parkinson’s Disease

The study design will be quasi-experimental with a control group, repeated measures and a combination of quantitative and qualitative methods. Figure 1 shows a schematic outline of the study design and Table 1 presents the project timeline. The baseline recruitment of participants and data collection started in March 2015 and is still ongoing (30% of the study still to be completed by the end of 2018).

**Table 1:** Project Timeline

	Year 1 (2015)			Year 2 (2016)			Year 3 (2017)			Year 4 (2018)		
Task	Q1	Q2	Q3	Q4	Q5	Q6	Q7	Q8	Q9	Q10	Q11	Q12
Random allocation of interventions to participating centres	×											
Dissemination of interventions in participating centres	×	×	×	×	×	×	×	×	×			
Delivery of letters to patients and carers	×		×		×		×		×			
Data collection at baseline (Questionnaire T0)	×		×		×		×		×			
Participants from experimental and control groups receive the intervention at a time	×		×		×		×		×			
Data collection after the intervention (Questionnaire T1)	×		×		×		×		×			
Data collection 6 months post-intervention (Questionnaire T2)			×		×		×		×		×	
Focus groups post-intervention with patients and carers					×	×	×					
Focus group with professionals										×		
Analysis of qualitative data					×	×	×	×	×	×	×	×
Analysis of quantitative data											×	×
Dissemination of findings											×	×

This study is part of the ReNACE research programme aimed at designing multidisciplinary and individualised interventions to promote positive living with a LTC in patients and relatives.

### Setting

The study will be carried out in several urban areas of Navarra, in the North of Spain. Specifically, the six health centres in a community setting with the largest numbers of people diagnosed with PD will be chosen in order to maximise the recruitment of participants and improve the power of the study. At the end of the recruitment of participants, ANOVA and χ^2^ tests will be performed to explore the sociodemographic characteristics of the participants from the intervention group and the control group. In the case of finding a significant difference in a sociodemographic variable, the statistics analyses will adjust by propensity-score analysis or the most suitable statistical technique.

### Participants

Participants will be people with PD receiving care as outpatients in the designated centres, over the age of 18 years, in any stage of the disease (as determined by a researcher with the Hoehn & Yahr scale [22]), residing at that time in Navarra, fluent in the speaking and understanding of the Spanish language, and with preserved cognitive ability (as determined by medical history or the doctor responsible).

Family members/carers will be over the age of 18, living or maintaining a close relationship with the patient and actively collaborating in his/her care, currently residing in Navarra and fluent in the speaking and understanding of the Spanish language.

Individuals with PD may enter the research project even though they do not have a family carer or the carer does not wish to participate. The family carer will also be able to enter the research project even though the individual with PD cannot participate for not meeting the inclusion criteria or for lack of interest.

Any change in participants’ circumstances during the study which mean that they do not meet the inclusion criteria mentioned above will cause the discontinuation of the participants in the study.

### Sample size

For the quantitative data collection, the sample size necessary to detect medium to large differences in the primary outcome of the project, quality of life, was calculated. To this end, a previous study of the ReNACE programme [11] with patients with PD and family carers was taken as a reference point together with previous studies [13, 23], using the quality of life scales that will be used in this study in people with PD and family carers. Furthermore, previous research on patients [19, 24] and family carers [25] has been taken into account to calculate the percentage of losses expected during follow-up. Calculation of the sample size was performed using the statistical programme STATA for both control and intervention groups. The parameters used for the calculation of the sample size are shown in Table 2.

**Table 2:** Parameters used for the calculation of the sample size by group

	Mean (SD) Quality of life	Size effect. Clinically significant difference	Power	Expected losses over follow-up	Ideal sample size
Patients	29.03 (15.44)^a^	10	80%	25%	52
Carers	104.70 (25.04)^b^	15	80%	20%	53

SD: standard deviation

^a^Questionnaire on Parkinson’s disease PDQ-39 of Peto et al. (1995) [26]

^b^Scale of Quality of Life of Care-givers SQLC of Glozman et al. (1998) [31]

The minimum sample size calculated is 52 patients and 53 family carers for the intervention group and the same for the control group. Therefore, the study will require a minimum of 104 patients with PD and 106 family carers.

### Recruitment

A consecutive cases sampling will take place in the centres participating in the study until the minimum calculated sample size is achieved (see above).

Given the difficulties involved in the recruitment of this population, different strategies have been planned [26] to reach the required sample size: a) Posters will be put up announcing the study in visible areas in the participating centres; b) Letters will be sent from the centres themselves to the homes of all the people diagnosed with PD who meet the inclusion criteria. The letters will contain information about the study and how to join the study, a stamped addressed envelope and the informed consent form; c) The study will be explained to the professionals of the participating centres in a session. Here they will be encouraged to personally inform the people who come to their consultations about the study; d) In the strategies described above the ReNACE logo will be included, which has been designed to distinguish the study from other information that the target population might receive. Those interested may sign up for the study by telephone, by sending a letter in a stamped addressed envelope (provided by the research team) or in person at the reception of the participating centres. Subsequently, a member of the research team will evaluate if the potential participants meet the inclusion criteria by means of a brief interview in the health centre. Once it has been established that participants fulfill the inclusion criteria, they will be given a document with the dates of the sessions that they must attend and of data collection.

### Procedure

There will be two groups in this study. Participants in group A will receive the ReNACE psychoeducational intervention while the participants in group B, belonging to the control group, will be given a general educational programme.

Allocation will be made according to the centre in which participants are attended. The experimental intervention was assigned to the centres in a random draw by tossing a coin (heads-control, tails-experimental) [27]. In this study individual randomisation, that is by patient or family carer, is not feasible because there is a high risk of contamination between intervention and control groups when participants interact with each other during their visits to their health centre. This is due to the patients from each health centre usually have the same family physician and nurse, and live in the same neighborhood. We decided to choose the six health centres with the highest numbers of people diagnosed with PD. With this proposed allocation strategy it is expected that contamination between the control and intervention groups will be minimal.

Furthermore, participants will be blinded to intervention assignment [28] as they will only be aware of the intervention they are receiving and they will not know if they belong to the intervention or control group. The healthcare professionals helping the participants in data collection will also be blinded to intervention assignment [28]. The healthcare professionals delivering the sessions to all the participants in the control and intervention groups will always be the same.

### The intervention

The group A will participate in the ReNACE psychoeducational intervention for 9 weeks with one 90 min group session per week. The objective of the contents of this intervention is to foster the coping skills of the participants to facilitate and promote their psychosocial adjustment to PD. The sessions will take place in groups of 15–20 people at most so as to promote reflection and facilitate the interchange of opinions between participants about issues of interest from their day to day experience of coping with PD. Table 3 shows the organisation of the topics that will be dealt with in each weekly session.

**Table 3:** Summary of the ReNACE psychoeducational intervention. Intervention group (A)

	Target audience	Topic	Provider
Session 1	Patients	Global introduction to the intervention	General practitioner
	Carers	Global introduction to the intervention	General practitioner
Session 2	Patients	Getting to know Parkinson’s disease	Neurologist
	Carers	Healthy life habits	Primary Care nurse
Session 3	Patients	Healthy life habits	Primary Care nurse
	Carers	Getting to know Parkinson’s disease	Neurologist
Session 4	Patients	Resources	Social worker
	Carers	Management of stress and complicated situations	Psychologist
Session 5	Patients	Adapting to and coping with Parkinson’s disease	General practitioner and Expert patient
	Carers	Resources	Social worker
Session 6	Patients	Positive self-esteem *&* Empathy and patience	Psychologist
	Carers	Look for information and live in the present *&* Normalise the situation and partake in activities	Psychologist
Session 7	Patients	Management of stress and complicated situations	Psychologist
	Carers	Positive self-esteem *&* Empathy and patience	Psychologist
Session 8	Patients	Look for information and live in the present *&* Normalise the situation and partake in activities	Psychologist
	Carers	Adapting to and coping with Parkinson’s disease	General practitioner and Expert patient
Session 9	Patients	Conclusions	General practitioner
	Carers	Conclusions	General practitioner

The main focus of the intervention is on empowering participants’ coping skills with stressful situations, which could clearly cause a crisis in the positive living with a LTC like PD [21]. This approach seeks that the participants become aware of their cognitive and behavioural efforts, think about them, and choose those that help them better adjust to PD [14]. In particular, professionals will work with the meaning of coping with PD explained by Navarta-Sánchez et al. [14]. All sessions will take place on the same day with the people with PD and their family carers, but in separate groups (see Table 3). This has been planned so as to prevent patients from not expressing themselves freely for fear of hurting the feelings of their family carers and vice versa. At the same time, as is shown in Table 3, the intervention will be delivered by a multidisciplinary team with years of clinical experience working with people with PD and other LTCs. All professionals of this team will use a manual which contains a description of the content and methodology of each session in order to improve adherence to intervention’s characteristics.

Furthermore, in one of the sessions a person with PD and a long history of the disease and a great interest in helping other people with PD to better cope with their situation will participate. Precisely with this objective, this person wrote a book, which will be offered for free as supportive written material to the participants. Also, in all the sessions participants will receive a document summarising the content covered and presented during the session on a projection screen.

The attendance of participants will be controlled in all sessions so that these data are available for analysis of the results. Those participants not attending at least 70% of the intervention sessions will be counted as lost to the study. Bearing in mind the complexity of the recruitment process and the duration of the interventions, it is expected that the interventions will need to be delivered over a period of 18 months.

### The control group

The general educational programme for the control group or group B (see Table 4) will last 5 weeks with a 90 min group session each week.

**Table 4:** Summary of the general educational program. Control group (B)

	Target audience	Topic	Provider
Session 1	Patients	Introduction	General practitioner
	Carers	Introduction	General practitioner
Session 2	Patients	Parkinson’s disease	Neurologist
	Carers	Habits for a healthy life	Primary Care nurse
Session 3	Patients	Habits for a healthy life	Primary Care nurse
	Carers	Parkinson’s disease	Neurologist
Session 4	Patients	Resources	Social worker
	Carers	Resources	Social worker
Session 5	Patients	Conclusions	General practitioner
	Carers	Conclusions	General practitioner

The objective of this programme is to provide participants with general information about PD, healthy lifestyles, and the resources available in the community. Through this intervention participants will be provided with the information generally received from social and healthcare professionals in usual care. The participants will receive this information in group sessions in order to simulate the effect of social interaction that may occur between the participants of the intervention group. It is estimated that the number of people attending each session will be between 15 and 20 at most. The issues that will be dealt with in each session are presented in Table 4. All sessions will take place on the same day with the patients and their family carers but in separate groups in separate rooms for the same reasons as described in the previous section. Attendance to the sessions will be registered as explained above.

### Outcome measures

#### Quantitative data collection

The primary outcome is the improvement in quality of life of the people with PD and of the family carers participating in the ReNACE psychoeducational intervention, in comparison with the change observed in the quality of life of those participating in the general educational programme. The secondary outcomes will be the changes in the coping skills and in the psychosocial adjustment to PD. These outcomes will be measured in the control and intervention groups at 3 time points; baseline (T0); immediately after the end of the intervention (T1); and 6 months post-intervention (T2). To ensure blinding of the groups and the intervention, researchers involved in data collection will not have participated in any of the group sessions and known the group allocation. At baseline (T0) the sociodemographic data of the participants will be collected. At the three time points the following instruments will be used to measure the outcomes:

Patients:

- The Quality of Life Scale in Parkinson’s disease (PDQ-39) [29, 30]
- The Psychosocial Adjustment to Illness scale (PAIS-SR) [31]
- The Brief COPE scale [32, 33]

Family carers:

- The Scale of Quality of Life of Care-givers (SQLC) [23, 34]
- The Psychosocial Adjustment to Illness scale (PAIS-SR) [31]
- The Brief COPE scale [32, 33]

The measurement scales selected are validated instruments which furthermore, have been used previously in a study of the ReNACE programme in PD patients and their family carers [11].

It is calculated that the participants will need approximately 1 hour to complete the questionnaires. When the participants finish the questionnaire, the researcher will check that they have been filled in correctly in order to minimise data loss.

To favor the collection of data at the most appropriate moment, participants will be given an appointment in their local centre to answer the questionnaires at T0 and T1. If participants do not attend these meetings they will be contacted by telephone to explain that the questionnaire will be sent by postal mail and that they should hand it in the centre or send it in the stamp addressed envelope within the period of 1 week. Furthermore, they will be given a telephone number that they can ring should they have any doubts about the questionnaire. For the evaluation of the results at T2 (6 months post-intervention) the questionnaires will be sent by post to all the participants and the same process as described above will be followed for their collection.

#### Qualitative data collection

Additionally, 2 focus groups will take place with patients and 2 focus groups will be developed with family carers. Another focus group with professionals delivering the intervention will be held.

The selection of patients and family carers will be made by means of intentional sampling with maximal variety [35], to promote the selection of participants from the intervention group of different ages, gender and number of years experience living with PD. These focus groups will take place 6 months after the ReNACE psychoeducational intervention was delivered. The aim of these focus groups is to explore the patients’ and family carers’ perceptions of the benefits of the ReNACE intervention in terms of coping skills, psychosocial adjustment and quality of life. Furthermore, participants will be asked their opinion on the issues covered and the methodology used. These focus groups will not be used to compare satisfaction with the interventions between groups (control versus experimental) because their goal is to understand better the changes related to the ways of coping of participants, that will be worked only in the ReNACE psychoeducational intervention. To this end a semi-structured interview guide will be used with questions such as the following displayed in Table 5.

**Table 5:** Questions to guide the focus groups of participants and healthcare professionals

Focus groups with people with Parkinson and family carers
• What do you think of the contents of the workshop?
• What do you think about the way in which the workshop took place?
• Could some aspects be improved?
• What have you got from participating in these workshops?
• How would you now rate your ability to cope with the disease?
Focus group with healthcare professionals
• What is your opinion of the content of the intervention?
• What do you think about the way in which the intervention took place?
• How do you think the information has been understood by the participants?
• What was the attitude of the participants in the intervention?
• How do you think the intervention may have influenced the psychosocial adjustment of the patients and family members?
• How might the sessions have changed the attitude of the participants to cope with the disease?
• How do you think could be the implementation of the intervention into care pathways?

The focus group with the social and healthcare professionals responsible for imparting the ReNACE psychoeducational intervention will be organised once the intervention has finished. The aim of this analysis is explore the professionals’ perspectives of the intervention and its integration as part of care pathways, their role in its future implementation, time needed and costs. A semi-structured interview guide will be used to guide the dialog around the questions shown in Table 5.

In all the focus groups voice recordings will be made which will subsequently be transcribed.

### Data analysis

#### Quantitative data

Analysis of quantitative data will be performed on an intention to treat basis and for that SPSS version 23.0 will be used. A descriptive analysis of the sociodemographic data will be made. For the quantitative variables, means and standard deviations will be calculated. Mixed ANOVA within-pairs and between-pairs to compare patients with family carers at each measurement points, unpaired student t tests to determine any significant differences between the two groups at baseline (T0) and the measurements at T1 and T2 will be used. Also, a mixed factorial ANOVA with 1 between-subjects factor with 2 levels (intervention and control), and 1 within-subjects factor with 3 levels (baseline, time 1 post-intervention, time 2 post-intervention) will be used to compare differences between the two groups in the main variables over time. If any demographic variable result different will be use as a covariate in this analysis.

#### Qualitative data

An in-depth analysis will be made of the transcriptions of the focal groups using content analysis [35]. The Nvivo® programme will be used to facilitate the handling and storage of the data. Analysis of the data will begin after the first focus group has been organised so that the results will help to enrich successive data collection. Analysis of the data will be performed individually by two researchers who will then meet to compare codes, categories and themes identified and to agree on the final results.

### Ethical considerations

Ethical approval for this study was obtained from the Ethics Committee of the University of Navarre in May 2014. After this, approvals from the participating centres were gained. Participants will sign an informed consent form that state their decision to take part in the study is voluntary, do not affect to their healthcare and that they can leave the study at any time. All personal information will be kept confidential. This information will be explained by a researcher to the participants in order to solve any doubt.

### Validity and reliability

The contents of the ReNACE psychoeducational intervention have been designed on the basis of previous research [11, 14, 21, 36, 37] carried out by the ReNACE programme team. This research highlighted the relevance that coping and adjustment have for living with PD in a positive way, both in patients and family carers. It further underlined the influence that exists between coping and adjustment and in turn between adjustment to PD and quality of life in this population [11, 18]. Furthermore, the present study will be conducted according to the philosophy of the Chronic Care Model [38]. Therefore, the strengthening of trust and motivation in participants for taking care of their health and coping skills will be an essential feature of the psychoeducational intervention. This study follows the recommendations of SPIRIT in the protocol and of the World Health Organization in the trial registration data. So as the qualitative methods of the study, researcher triangulation to validate the analysis of the data will take place as described [39].

## Discussion

This article describes the protocol of a quasi-experimental study with a control group and mixed methods designed to determine the effectiveness of a multidisciplinary psychoeducational intervention aimed at improving the quality of life of people with PD and their carers.

In the literature some non-pharmacological interventions have been identified [13, 19, 20, 24, 40–42] which sought to enable people with PD to deal with the different consequences of living with their illness. Although, in only two interventions [19, 20] were the development of coping skills and improvement in the psychosocial adjustment to PD promoted, no measurement was made of the changes occurring in these aspects. In accordance with the experience provided by these previous interventions [13, 19, 20, 24, 40–42], it is foreseen that the present quasi-experiment will be able to be carried out adequately in the target population.

Furthermore, as in the majority of previous interventions [20, 24, 40, 42], it is expected that with the ReNACE psychoeducational intervention statistically significant results will be obtained in the short-term, immediately after the end of the intervention. As for the evaluation of long-term results, in most of the studies conducted no statistically significant long-term results were obtained [20, 42] or they were not evaluated [19, 24, 41]. This shows the complexity involved in enabling people with PD to continue coping with the new situations that they will encounter when living with this neurodegenerative disease once the intervention period has finished but the progression of the disease and its consequences continues. However, the results of two randomised controlled trials into PD [13, 40] and a systematic review in LTCs [43] suggest that interventions focusing on improving lifestyles, redefinition of biography, social networks, emotional wellbeing and activities of daily life, such as the ReNACE intervention (see Table 3), represent tools with a great potential to help people with LTCs to know in the long-term how to deal with the difficulties caused by living with the disease. Specifically, considering the dynamic nature of the process of coping and adjustment to PD, this is where Primary Care professionals could play a vital role because if we demonstrate that these types of interventions are effective and feasible through projects, its continuation and reinforcement from a clinical perspective in the community will be the next step for the long term sustainability of the improvements and transformation of care pathways.

Furthermore, it is worth noting the small number of studies that have taken into account the need to train the family carers of patients with PD. However, there is an increasing number of authors [9–11, 36] who underline the importance of engaging family carers in a training process that allows them to better cope with the experience of the LTC. For this reason, in the quasi-experiment that we propose, the family carers will receive sessions with information suitable for their needs and this will clearly contribute to one of the big gaps in the literature when it comes to educating and caring from PD patients and family carers as a family unit.

The present quasi-experimental study aims to demonstrate the benefits that patients with PD and their family carers can obtain if they receive psychosocial care oriented towards health promotion. However, the ultimate goal of this research is to achieve the full incorporation of the proposed psychoeducational intervention into the daily practice of the different social and healthcare professionals that interact with patients with PD and their family members.

Consequently, this study although at a small scale constitutes an important research and conceptual leap in the available literature, which lacks evidence of interventions for people with PD and family carers as a whole and seeks an approximation between the research and the clinical reality in Primary Care. Furthermore, the psychosocial nature of the proposed intervention approaches a neglected area in research and practice, which will clearly contribute to reduce the burden of PD for patients and family carers and the existing knowledge [44, 45].

It is expected that the results of the study will contribute to the creation of similar psychoeducational interventions for people with other LTCs and their family carers. In the long-term it is hoped that this type of intervention will help to consolidate a sustainable care network, which will fully cover the needs of people with LTCs and their families thus favoring their emotional wellbeing, social life, biography and a healthy lifestyle.

### Limitations

The non-randomised and voluntary nature of the participation could limit the generalisation of results. However, statistical generalization is not sought in this study but a breakthrough in an area of interventional research that has been neglected for many years when it comes to PD and family carers and illness adjustment. In particular, patients at an advanced stage of PD may not take part in the intervention due to their disability and that young patients who have been diagnosed only recently may not enroll in the intervention due to the stigma of PD being thought of as a disease of the elderly. However, given that centres from different health areas of Navarra will participate, it is expected that the likelihood of this effect influencing the results will be diminished. Similarly, the reliability and sensitivity of the measurement scales that will be used in the study, together with the use of repeated measures, the presence of a control group and the baseline comparison between groups, are all important factors that will help to overcome the limitation mentioned above.

Another limitation could be the baseline differences between the centres selected in the quasi-experiment. However, it is expected that non-statistically significant differences will be found between participants given that the populations of the centres share a similar socio-economic and cultural context.

Furthermore, a limitation of this study is to successfully encourage people with PD and their family carers, who generally do not participate in activities on a regular basis, to attend the intervention each week. It is necessary to bear in mind that given the age and physical condition of the target population the number of subjects lost to the study could be greater than usual in longitudinal studies. However, to minimise this effect, at the end of each session participants will receive a small introduction to the topic of the following week which will help to increase enthusiasm and promote regular attendance.

It is also necessary to point out that in this study it is not possible for the professionals giving the sessions to be blinded, given that there are two different types of intervention. However, researchers involved in the data collection and participants for group A and B will be blinded to the intervention. Also the content and methodology of each session will be established previously in a manual that the professionals will use.

A final potential limitation of this study is that two types of data collection will be used. At measurement points T0 (baseline) and T1 (post-intervention) all participants will be given an appointment at their health centre to fill in the questionnaire the same day. In contrast, at measurement point T2 (6 months post-intervention) participants will receive the questionnaire by post and will fill it in in their homes. Nevertheless, given that the participants will have completed the questionnaire on two previous occasions no difficulties are expected for completing the questionnaire at measurement point T2 and doubts will be solved when needed. Moreover, to avoid high numbers of participants not returning or forgetting returning the questionnaire at T2, health professionals from the health centres will be involved in the follow up.

## Conclusions

Currently, in Primary Care it exists the need to implement psychoeducational interventions to promote coping skills and the psychosocial adjustment to PD not only for patients but also for family carers. Such interventions form an essential part of the development of a more holistic approach in clinical practice, focused not on the disease but on the person and his or her experience of the disease which furthermore would respond to the concerns and problems of the family carers. Therefore, the aim of this study is to also promote a change in social and healthcare policies by favoring the introduction of this psychoeducational and health promotion approach into the clinical practice of Primary Care for the management of PD and other LTCs.

## Abbreviations

LTCs: Long Term Conditions
PD: Parkinson’s disease
